# Electrochemical and Infrared Absorption Spectroscopy Detection of SF_6_ Decomposition Products

**DOI:** 10.3390/s17112627

**Published:** 2017-11-15

**Authors:** Ming Dong, Chongxing Zhang, Ming Ren, Ricardo Albarracín, Rixin Ye

**Affiliations:** 1 State Key Laboratory of Electrical Insulation for Power Equipment, Xi’an Jiaotong University, Xi’an 710049, China; dongming@mail.xjtu.edu.cn (M.D.); zhangcx.123@stu.xjtu.edu.cn (C.Z.); 2 Departamento de Ingeniería Eléctrica, Electrónica, Automática y Física Aplicada, Escuela Técnica Superior de Ingeniería y Diseño Industrial (ETSIDI), Universidad Politécnica de Madrid (UPM), Ronda de Valencia 3, Madrid 28012, Spain; rasbarracin@gmail.com; 3 Maintenance Company of State Grid Shaanxi Electric Power Company, Xi’an 710065, China; yexiaowen213@163.com

**Keywords:** SF_6_ gas decomposition products, electrochemical sensing (ES), infrared (IR) spectroscopy, detection characteristic, detector

## Abstract

Sulfur hexafluoride (SF_6_) gas-insulated electrical equipment is widely used in high-voltage (HV) and extra-high-voltage (EHV) power systems. Partial discharge (PD) and local heating can occur in the electrical equipment because of insulation faults, which results in SF_6_ decomposition and ultimately generates several types of decomposition products. These SF_6_ decomposition products can be qualitatively and quantitatively detected with relevant detection methods, and such detection contributes to diagnosing the internal faults and evaluating the security risks of the equipment. At present, multiple detection methods exist for analyzing the SF_6_ decomposition products, and electrochemical sensing (ES) and infrared (IR) spectroscopy are well suited for application in online detection. In this study, the combination of ES with IR spectroscopy is used to detect SF_6_ gas decomposition. First, the characteristics of these two detection methods are studied, and the data analysis matrix is established. Then, a qualitative and quantitative analysis ES-IR model is established by adopting a two-step approach. A SF_6_ decomposition detector is designed and manufactured by combining an electrochemical sensor and IR spectroscopy technology. The detector is used to detect SF_6_ gas decomposition and is verified to reliably and accurately detect the gas components and concentrations.

## 1. Introduction

Electrical equipment is the fundamental basis of power systems, and its reliability is crucial to ensure grid security and stability [1]. With their many advantages, such as a compact structure, stable insulation performance, strong flowing-breaking ability, and reliable operation, SF_6_ gas-insulated electrical equipment, especially gas-insulated-switchgear (GIS) and gas-insulated-transmission line (GIL), has been widely applied in high-voltage (HV) and extra-high-voltage (EHV) power systems worldwide [2]. However, partial discharge (PD) and local overheating can occur in the GIS because of insulation faults, causing SF_6_ gas decomposition and ultimately generating several types of SF_6_ decomposition products, including SO_2_F_2_, SOF_2_, CO, SO_2_, H_2_S, HF, CF_4_, and SiF_4_ [3,4]. These decomposition products have a severe effect on the insulating materials, accelerate insulation deterioration in the GIS, and can even result in equipment damage. Once the GIS insulation fails, the state-of-safe and reliable GIS operation faces a severe threat. The variation of these gases reflects the equipment internal PD and insulation status. Through quantitatively detecting and analyzing these gaseous components, potential equipment failure and security problems can be diagnosed to provide an early warning of degraded device status [5,6,7,8].

The analysis techniques for SF_6_ decomposition are an important method for diagnosing the insulation condition of SF_6_ gas-insulated electrical equipment. Relative to other detection methods, the SF_6_ decomposition detection methods have many advantages, including resistance against jamming, high-sensitivity and quantitative analysis. Furthermore, with the existence and development of equipment internal defects, the amount of SF_6_ decomposition products can gradually accumulate; thus, this method is particularly suitable for long-term monitoring. Substantial worldwide research has been directed to the detection of SF_6_ decomposition products. These detection methods are used to analyze the components and concentrations of such products and ultimately to deduce the partial discharge type and severity. The methods include gas chromatography, mass spectrometry, infrared (IR) spectroscopy, electrochemical sensing (ES), gas detector tubes, ion mobility spectrometry, and carbon nanotubes, among other methods [9,10,11,12,13]. However, these detection methods still generally depend on regular field sampling and complete quantitative analysis of SF_6_ decomposition products in the laboratory. Relative to other detection methods, electrochemical sensors and IR spectroscopy offer particularly favorable prospects for online detection [9,14,15]. Therefore, it is necessary a further investigation in electrochemical sensors and IR spectroscopy for detecting SF_6_ decomposition products, which is the focus of this study.

Here, by first establishing a testing platform of ES and IR spectroscopy, we obtain the response characteristics of the electrochemical sensor and the spectral characteristic of target detection gases (CO, SO_2_ and H_2_S), including the temperature, temperature compensation and linearity. Then, a qualitative and quantitative analysis model of ES-IR is established by adopting a two-step approach. Furthermore, a SF_6_ decomposition detector has been designed by combining electrochemical sensors and IR spectroscopy to lay the foundation for SF_6_ decomposition product online monitoring with an ES-IR evaluation of SF_6_ gas-insulated electrical equipment.

## 2. Detection Principle

### 2.1. Electrochemical Sensing

With advantages such as a stable working performance, long-service life, low-power consumption, high-sensitivity and rapid-response speed, the electrometrical gas sensor is well-suited for field quantitative detection of gases that are toxic at low concentrations and combustible gases [9,16]. ES also offers advantages such as small volume, simple operation and low cost, and has been widely used to conduct online monitoring in multiple fields, such as petrochemical industry, home automation, air quality monitoring, etc.

**Figure 1:**
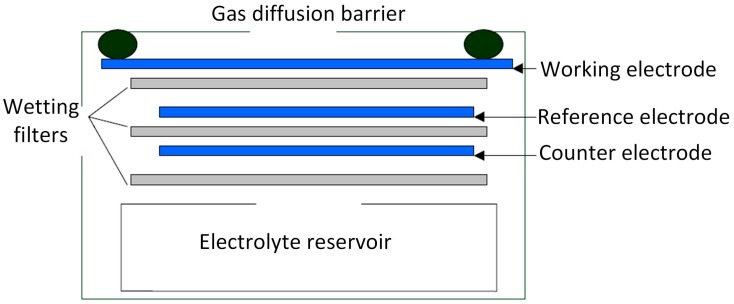
Schematic diagram of the electrochemical gas sensor.

Electrochemical sensors are electrochemical cells that operate in the amperometric mode. That is, sensors generate a current that is linearly proportional to the fractional volume of the target gas, such as CO, SO_2_ or H_2_S. The structure of an electrochemical sensor is shown in Figure 1. The sensor typically consists of three electrodes immersed in an electrolyte: the working electrode (WE), the counter electrode (CE) and the reference electrode (RE). The working principle of an electrode sensor is that the target gas diffuses into the WE surface by crossing a gas diffusion barrier, the WE either oxidizes or reduces the target gas with the CE balancing the generated current, and an external circuit outputs a current linearly proportional to the measured gas concentration. Taking the reaction of CO electrochemical sensor as an example, when the sensor works, CO is oxidized on the WE and O_2_ is reduced on the CE. And the reaction equation is shown as follows:

(1) $$\begin{array}{l} \mathrm{Oxidation}\;\mathrm{reaction}(\mathrm{WE}):\mathrm{CO}+H_{2}O\to {\mathrm{CO}}_{2}+2H^{+}+2e^{-}\\ \mathrm{Reduction}\;\mathrm{reaction}(\mathrm{CE}):\frac{1}{2}O_{2}+2H^{+}+2e^{-}\to H_{2}O\\ \;\mathrm{Overall}\;\mathrm{reaction}:\;\mathrm{CO}+\frac{1}{2}O_{2}\to {\mathrm{CO}}_{2} \end{array}$$

When the electrode potential and the catalytic activity are high enough, CO gas molecules penetrate the diffusion barrier close to the electrode and reacted rapidly. Then, the amplitude of the generated current is proportional to the CO gas concentration based on Fick’s law. Therefore, by measuring the current between WE and CE, the CO gas concentration can be obtained. Similarly, the total redox reaction of H_2_S and SO_2_ electrochemical sensors is expressed by the following equations:

(2) $$\begin{array}{l} {\mathrm{SO}}_{2}+H_{2}O+\frac{1}{2}O_{2}\to H_{2}{\mathrm{SO}}_{4}\\ H_{2}S+2O_{2}\to H_{2}{\mathrm{SO}}_{4} \end{array}$$

In reality, the WE surface always undergoes a continuous electrochemical reaction, which may result in sensor performance degradation. Thus, the RE is added to improve sensor performance. The RE anchors the WE at the correct potential to ensure that the reaction stays in the transport-limited current plateau of the current-voltage curve.

### 2.2. IR Spectroscopy

Material molecular movement follows the law of quantum mechanics. According to the Born-Oppenheimer approximation of quantum mechanics [17], the energy of molecular motion (*E*) is composed of translational (*E_t_*), rotational (*E_r_*), vibrational (*E_v_*) and electronic energy (*E_e_*). Thus, the energy of molecular motion can be expressed as follows:

(3) $$E=E_{t}+E_{r}+E_{v}+E_{e}$$

The energy difference of adjacent energy levels of a molecule’s translational kinetic energy is relatively small and can be regarded as changing continuously. The rotational, vibrational and electronic transitions are all quantized. When using IR to detect gas samples, the molecule undergoes an energy level transition from a lower energy level *E*_1_ to a higher energy level *E*_2_. The gas sample can selectively absorb the specific frequency of IR light that satisfies the following equation:

(4) $$\Delta E=E_{2}-E_{1}=hv$$

where *h* = 6.624 × 10^−34^ J·s and *v* is the frequency. IR absorption spectroscopy exploits the fact that molecules can selectively absorb electromagnetic (EM) radiation in the IR zone. Each type of molecule absorbs wavelengths in a unique IR spectrum corresponding to its molecular vibrational and rotational frequency. Thus, the IR absorption spectrum of a gaseous decomposition product features a peak at the gas absorption wavelength called the typical absorption peak for this gas. Through analyzing the typical absorption position, value and shape of the gas sample IR spectrum, the decomposition products of SF_6_, for example, can be qualitatively and quantitatively detected [7].

Specifically, IR absorption spectroscopy for quantitative detection is based on the Lambert-Beer Law [18], which states that the gas sample absorbs a specific frequency of IR light when a beam of light passes through the gas sample; the absorption intensity is proportional to each component concentration and optical path length [19]. For the IR absorption spectra of a single gas sample, the absorbance at any wave number (*v*) is expressed as follows:

(5) $$A\left(v\right)=\mathrm{lg}\frac{1}{T\left(v\right)}=a\left(v\right)bc$$

where the dimensionless parameter *A*(*v*) is the absorbance, the dimensionless parameter *T*(*v*) is the transmittance, *a*(*v*) is the absorbance coefficient at the wave number (*v*) and its units are (kPa × μL/L × m)^−1^, *b* is the optical path length (unit: m), and *c* is the gas concentration (units: kPa × μL/L).

## 3. Testing Platform

**Figure 2:**
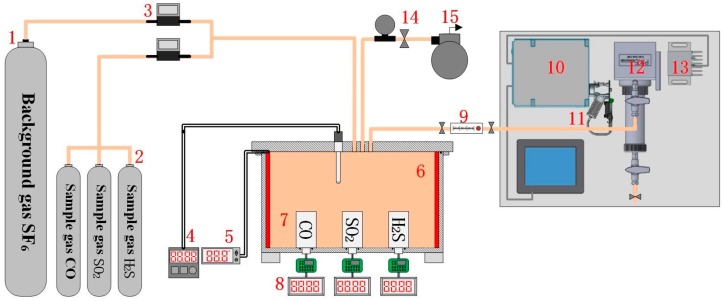
Test platform for electrochemical sensing. The label in the picture is indicated respectively as follows: 1. Background gas SF_6_; 2. Sample gas CO, SO_2_, H_2_S; 3. Mass flow controller; 4. Thermocouple; 5. Heating plate; 6. SF_6_ decomposition detection tank; 7. Electrochemical sensor; 8. Digital ammeter; 9. Glass rotameter; 10. Interferometer; 11. IR source; 12. Gas pool; 13. Detector; 14. Barometer; 15. Vacuum pump.

To obtain the response of the electrochemical sensor and spectral characteristics of the target detection gas, an experimental platform is first configured, as shown in Figure 2. The platform comprises a gas configuration system and a measurement system, including the background SF_6_ gas, the sample gases (CO, SO_2_, H_2_S), a mass flow controller, a SF_6_ decomposition detection tank, electrochemical sensors, and a Fourier transform infrared spectroscopy (FTIR) detection system.

The experimental gas configuration system consists primarily of background SF_6_ gas, sample gas, a mass flow controller, and a SF_6_ decomposition detection tank. The gas flow controller can be used to accurately control the gas volume, and various species and concentrations of experimental gas can be configured in the SF_6_ decomposition detection tank.

**Figure 3:**
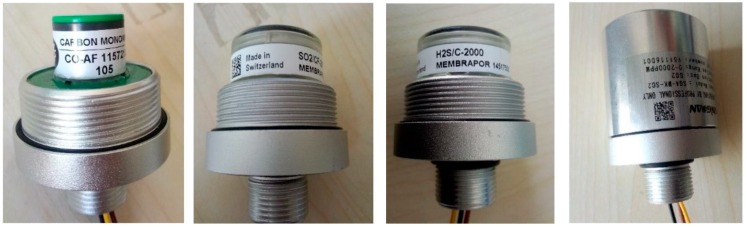
Electrochemical sensors.

The measurement system for sensor response contains the electrochemical sensors (see Figure 3), a digital ammeter, a heating plate and a thermocouple. The sensors that employ a threaded connection for ease of replacement is placed at the bottom of the tank and its main technical parameters is shown in Table A1. The heating plate is used to heat the experimental gas in the tank, and the thermocouple measures the ambient temperature inside the tank in real time, facilitating study of the sensor’s temperature characteristics. Through configuring various components and concentrations of the experimental gas, the sensor linearity characteristics can also be obtained.

**Figure 4:**
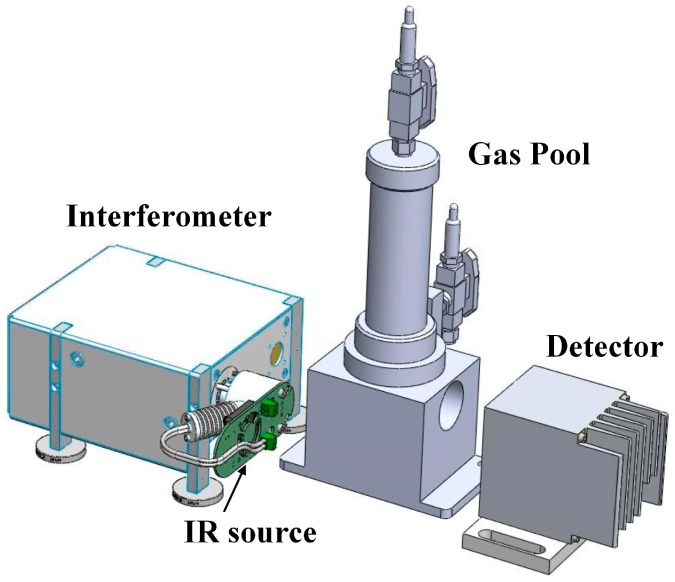
SF_6_ decomposition product detection system.

The FTIR detection system consists of an IR source, an interferometer module, a detector module and the gas pool, as shown in Figure 4 and Table A2. The IR source can emit 1–25 μm wavelength light, and the wavelength range can cover all the characteristic wavelengths for all measured gas components. The interferometer module is the key component and mediates the splitting and scanning of the IR light. The detector module is used to analyze the interference light containing the information from the measured gas; the model then obtains the gas component concentrations through IR spectroscopy.

## 4. Response Characteristics of the Sensors

### 4.1. Temperature Characteristics

**Figure 5:**
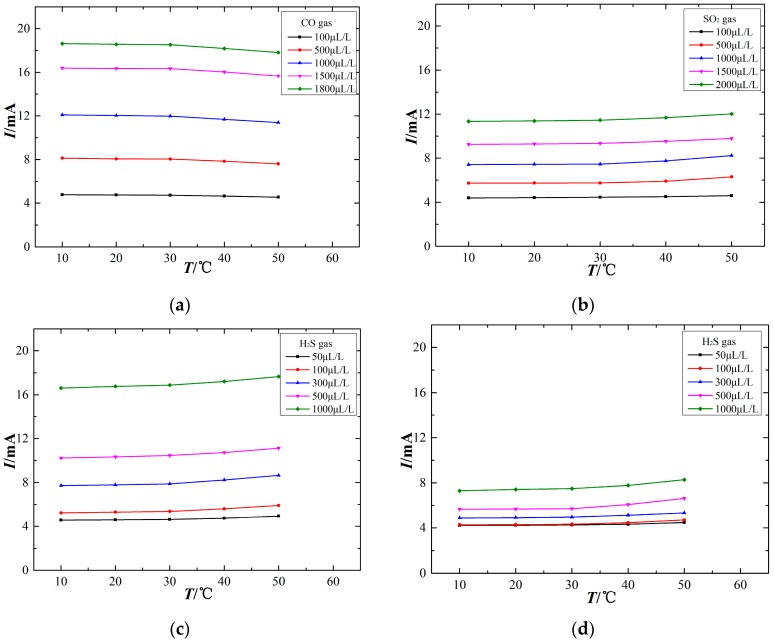
Temperature characteristics of the electrochemical sensors. (**a**) CO sensor, (**b**) SO_2_ sensor, (**c**) H_2_S sensor, (**d**) SO_2_ sensor (exposed to H_2_S).

The output signal of electrochemical sensors is closely related to the ambient temperature. To study the relationship between the sensor output signal and ambient temperature, the experiments are processed at different temperatures of 10 °C, 20 °C, 30 °C, 40 °C and 50 °C using sensors to detect the experimental gas. The temperature characteristics are shown in Figure 5.

The variation of ambient temperature affects the output signal of electrochemical sensors at a constant gas concentration. However, different gas sensors exhibit differences in their temperature-dependent response, as shown in Figure 5. Figure 5a shows that the output current signal of the CO electrochemical sensor decreases with increasing ambient temperature, although this temperature sensitivity is nearly absent over the range of 10–30 °C. Figure 5b,c show that the output signals of the SO_2_ and H_2_S sensors increase as the ambient temperature increases, and the higher the ambient temperature, the greater the rate of change for the output current signal. Figure 5d shows that the SO_2_ sensor also responds to H_2_S gas and that its output signal is positively correlated with ambient temperature. The sensor output signal is relatively stable at approximately 20 °C, which demonstrates that 20 °C may be an appropriate testing temperature for the sensors. When the ambient temperature is too high, the output signal exhibits a greater change. That is, a higher ambient temperature has a stronger effect on these electrochemical sensors. Therefore, such temperature-dependence sensors are difficult to be used directly for field detection.

The relationship between the sensor output signal and ambient temperature is influenced in two ways. First, the electrochemical sensors have an internal electrochemical reaction, and increasing the ambient temperature can enhance the reaction speed, thus increasing the output signal. Second, a gas diffusion barrier exists between the sensor and the external environment. With the use of various materials and fabrication processes, different sensor films exhibit differences in diffusion permeability, and the variation of ambient temperature influences the transport of gas molecules through the diffusion film. An increasing ambient temperature causes more energetic thermal motion of gas molecules, thereby affecting the gas contacting the working electrode surface, which may influence the sensor output signal. As a result, an increasing ambient temperature may not necessarily lead to an increased sensor output signal. It is important when using electrochemical sensors to ensure an optimal operating temperature over long experimental durations, and temperature compensation is necessarily adopted to suppress the effect of ambient temperature insofar as it influence the sensor measurements.

### 4.2. Temperature Compensation

**Figure 6:**
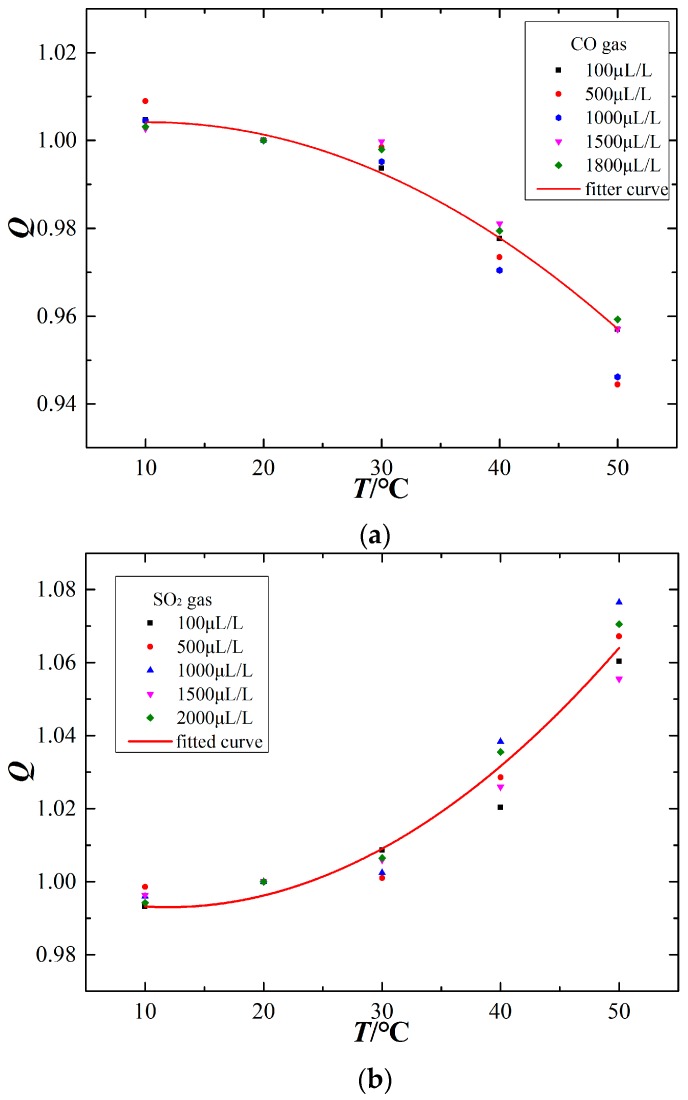

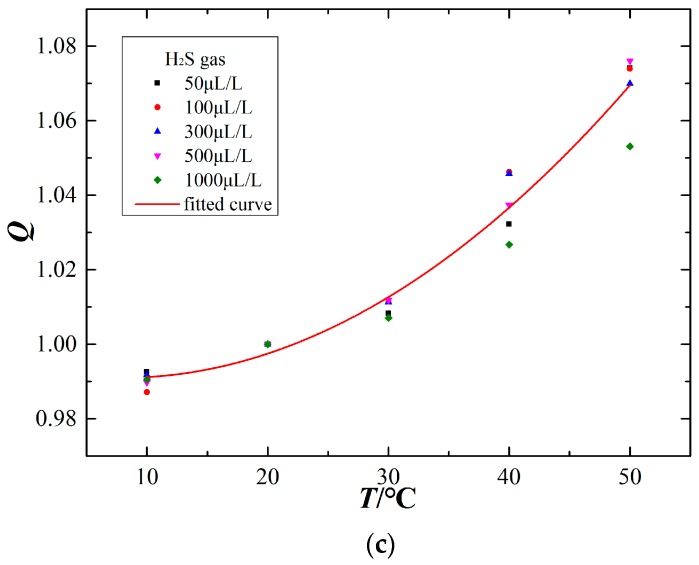
Temperature compensation curves of the electrochemical sensors. (**a**) CO sensor, (**b**) SO_2_ sensor, (**c**) H_2_S sensor.

To further study the relationship between the ambient temperature and sensor output signal, the temperature compensation curves are fit using a quadratic fitting method, as shown in Figure 6. The x-axis represents the ambient temperature (°C), the sensor output signal at an ambient temperature of 20 °C is chosen as the reference signal, and the *Q* of the y-axis represents the ratio of output signals at the measuring temperature and at 20 °C, as shown in the following equation:

(6) $$Q=\frac{I_{x}}{I_{20{}^{\circ}C}}$$

where *I_x_* is the measured output signals value; *I*_20°C_ is the measured output signals value at 20 °C.

The CO electrochemical sensor has a negative temperature sensitivity, whereas the SO_2_ and H_2_S sensors have a positive temperature sensitivity. The temperature compensation curves for the different sensors are as follows:

(7) $$\begin{array}{l} Q_{\mathrm{CO}}=f_{1}(T)=1.00101+0.0006123T-0.00002980T^{2}\\ Q_{{\mathrm{SO}}_{2}}=f_{2}(T)=1.01002-0.0011700T+0.00004893T^{2}\\ Q_{H_{2}S}=f_{3}(T)=0.99371-0.0006948T+0.00004211T^{2} \end{array}$$

where *T* is the ambient temperature during the measurement. Therefore, after the data normalization preprocessing, the actual output signals (*I_x_*) at the ambient temperature can be converted to the standard value (*I*_20°C_) at 20 °C by the following equation:

(8) $$I_{20{}^{\circ}C}=\frac{I_{x}}{Q}=\frac{I_{x}}{f_{i}(T)}$$

### 4.3. Linearity Characteristics

**Figure 7:**
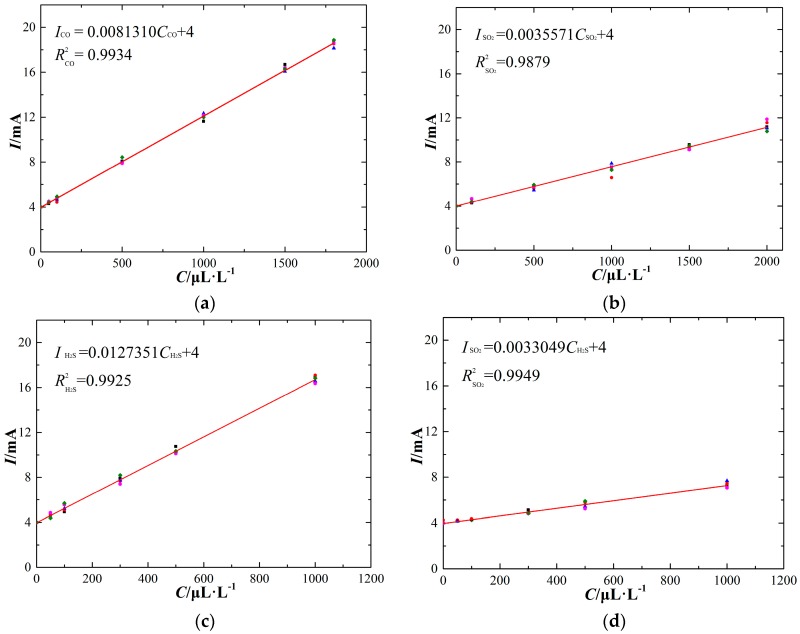
Linearity characteristics of the electrochemical sensors (at 20 °C). (**a**) CO sensor, (**b**) SO_2_ sensor, (**c**) H_2_S sensor, (**d**) SO_2_ sensor (exposed to H_2_S).

The electrochemical sensor generates a current signal linearly proportional to the target gas concentration. Through configuring various components and concentrations of experimental gas in the detection tank at one atmosphere and 20 °C ambient temperature, the linearity characteristics of the electrochemical sensors are obtained, as shown in Figure 7.

The experimental results show that the output current signal of different types of electrochemical sensors responds linearly with the corresponding target gas concentration. In addition, the SO_2_ sensor responds linearly to increasing H_2_S gas concentration. The sensor response can be fit to a linear equation between the output signal (*I*) and the target gas concentration (*C*):

(9) I=mC+n

where *m*, *n* is the slope and vertical intercept of the fitting line respectively. The sensor linearity characteristics are shown in Table 1.

Based on the linearity characteristics of these electrochemical sensors, the theoretical detection precision and measurement uncertainty can be analyzed. The sensor detection precision is an important parameter for evaluating detection performance, and the target output current precision of the sensors is two valid digits after the decimal point in units of mA. Therefore, the ideal minimum gas concentration detection of sensors can be calculated by assuming a sensor output current value of 4.01 mA in the linear formulas. The measurement uncertainty is also an important parameter. The upper envelope is expressed by the following equation:

(10) $$I_{max}=m_{max}C+n$$

which can be fit by choosing the maximum measurement value at each gas concentration. The lower envelope is expressed by the following equation:

(11) $$I_{min}=m_{min}C+n$$

which can be fit by choosing the minimum measurement value at each gas concentration. Comparing the two envelope lines with the optimal linearity characteristic in Figure 7, the measurement uncertainty can be calculated, as shown in Table 2.

### 4.4. Sensor Data Analysis Matrix

The working principle of electrochemical sensors can cause the sensor to respond to several types of gas with similar chemical properties. We determined that the SO_2_ sensor responds to SO_2_ and H_2_S gas. To accurately obtain the target gas concentration, the temperature compensation and crossing calculation are adopted, and the sensor data matrix is established [20]. The sensor data matrix between the real value of target gas concentration and the measured output current signal is as follows:

(12) $$I_{1}=QI=AC+b$$

where diagonal matrix ***Q*** = diag(*Q*_CO_, *Q*_SO_2__, *Q*_H_2_S_) represents the sensor temperature compensation coefficient matrix, ***I*_1_** = [*I*_1CO_, *I*_1SO_2__, *I*_1H_2_S_]*^T^* represents the sensor actual output signal matrix, ***I*** = [*I*_CO_, *I*_SO_2__, *I*_H_2_S_]*^T^* is the sensor output signal matrix at 20 °C, ***A*** = {*α*_ij_}(1 ≤ *i* ≤ 3, 1 ≤ *j* ≤ 3) represents the sensor crossing calculation matrix, ***C*** = [*C*_CO_, *C*_SO_2__, *C*_H_2_S_]*^T^* is the actual target gas concentration matrix, and ***b*** = [4,4,4] is the sensor output signal in pure SF_6_ gas.

Matrices ***Q*** and ***A*** can be obtained through a series of experiments, and then the actual target gas concentration can be obtained through matrix inversion:

(13) $$C=A^{-1}\left(I-b\right)=A^{-1}(Q^{-1}I_{1}-b)$$

A multi-component gas mixture is used to demonstrate the reliability and accuracy of the sensor data matrix. We configured the system to detect the multi-components with background SF_6_ gas and the target gases of CO, SO_2_ and H_2_S, and the experimental results are shown in Table 3.

The experimental results show that the measurement uncertainty of the CO sensor is less than 2% and that the measurement uncertainty of the SO_2_ and H_2_S sensors is less than 3%. The results show that the sensor data matrix method offers high reliability and accuracy.

## 5. Spectral Characteristic of the Target Gas

### 5.1. Spectral Simulation

**Figure 8:**
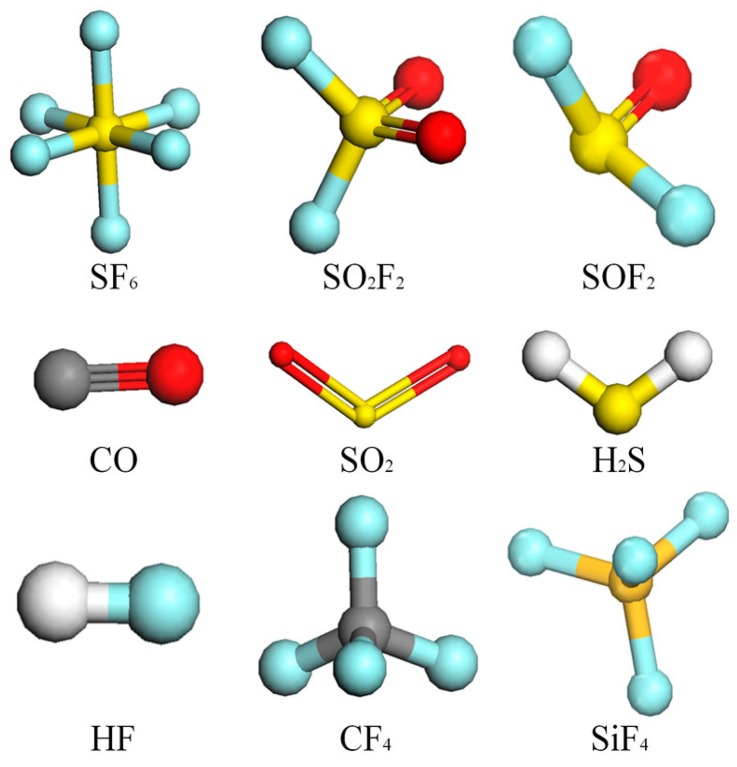
Molecular models of the decomposition products of SF_6_.

Material studio is used to simulate the IR absorption spectrum of SF_6_ and its decomposition products based on the molecular dynamics method [21]. The microstructure of different molecular molecules are first determined, as shown in Figure 8.

**Figure 9:**
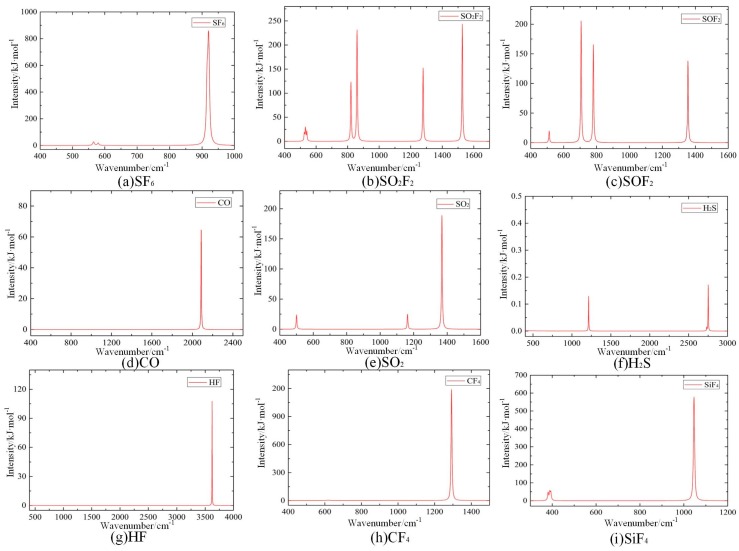
IR absorption spectroscopy of SF_6_ and its decomposition products.

The calculated IR absorption spectroscopy of SF_6_ and its decomposition products is simulated, as shown in Figure 9. The simulation results show that the IR absorption of SF_6_ and its decomposition products is concentrated predominantly within the range of 300–4000 cm^−1^. Different gas molecules have different IR absorption peaks, and their corresponding absorption intensities also exhibit a large difference. In addition, a single gas molecule can have multiple absorption peaks.

**Figure 10:**
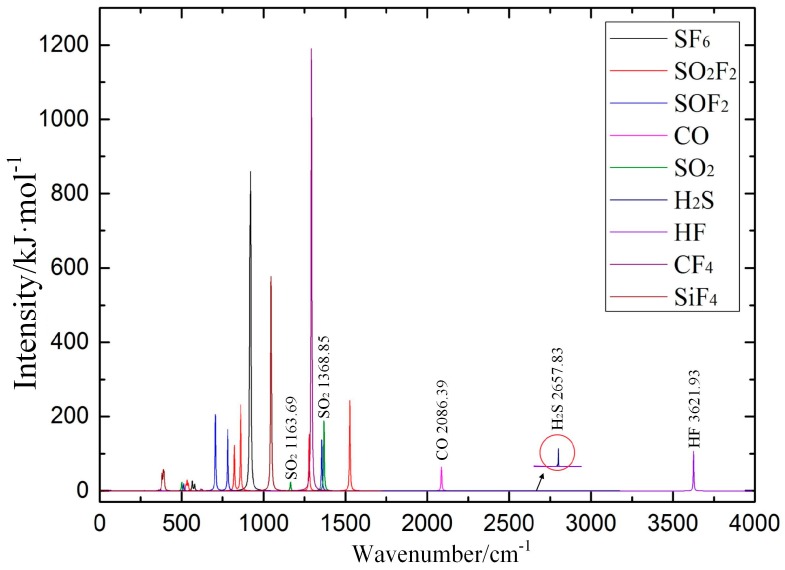
Calculated IR absorption spectroscopic results of SF_6_ decomposition products.

Polyatomic molecules can have a variety of vibration modes that correspond to several IR absorption lines of different intensity; thus, it is necessary to determine the characteristic IR peaks to identify the SF_6_ decomposition products. The calculated IR absorption spectroscopy is obtained by superimposing the IR absorption spectroscopy of SF_6_ and its decomposition products, as shown in Figure 10. The position, intensity and shape of IR absorption characteristic peaks of these gas molecules differ substantially. The above characteristics of IR absorption peaks are used to qualitatively and quantitatively analyze the SF_6_ decomposition products.

Through reviewing the relevant international standards and literature, we compared the results of calculated IR spectroscopy and measured IR spectroscopy, as shown in Table A3. The comparison shows that both calculated and measured results are consistent. It is possible to determine the attribution of IR absorption peaks for the SF_6_ decomposition products.

### 5.2. Selection of a Typical Spectrum

**Figure 11:**
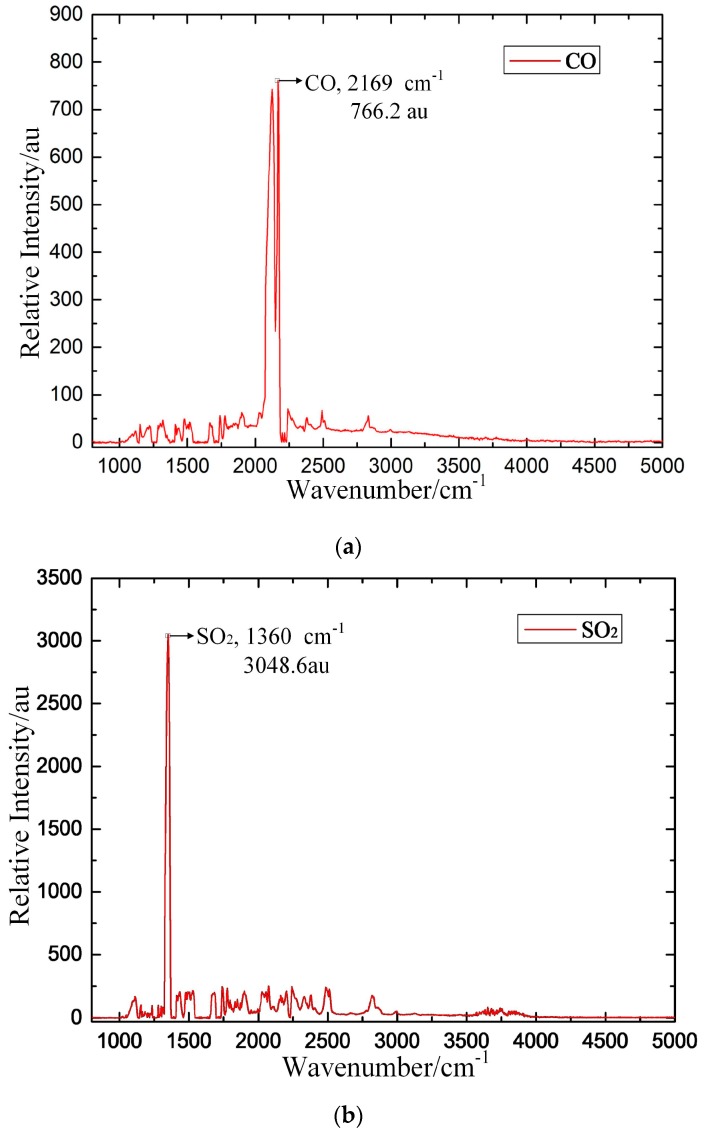

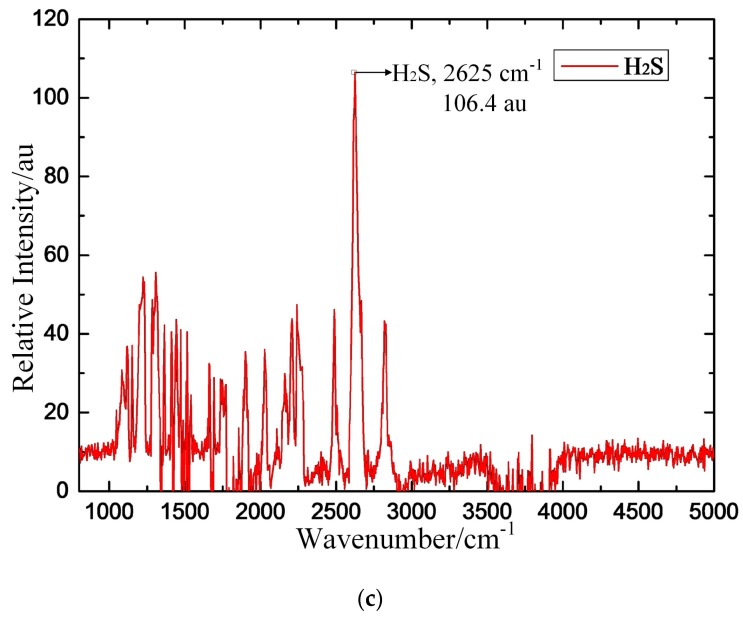
Measured IR spectroscopic results for the target detection gas. (**a**) CO, (**b**) SO_2_, (**c**) H_2_S.

The established FTIR detection system is intended to obtain the IR typical spectra of these three target gases. In the experiment, CO, SO_2_ and H_2_S are chosen as the target detection gases. Pure SF_6_ is the background gas, and the concentrations of CO/SF_6_, SO_2_/SF_6_, and H_2_S/SF_6_ are each 1000 μL/L. During the measurement, we ensure that the power supply and gas connection are safely and reliably connected. The gas pressure is adjusted to 0.1 MPa, and the measurement result is recorded after the gas flows for approximately 15 min. The experimental results are shown in Figure 11, in which au is the relative intensity of the IR spectrum.

The experimental results show that the IR spectra of CO, SO_2_ and H_2_S all contain prominent peaks. The relative intensity of CO is 766.2 au at the 2169 cm^−1^ characteristic peak, that of SO_2_ is 3048.6 au at the 1360 cm^−1^ characteristic peak, and that of H_2_S is 106.4 au at the 2625 cm^−1^ characteristic peak. The basic rules of characteristic IR line selection are as follows: (1) the characteristic IR line is typical for a single component in the mixture; (2) a higher peak is correlated with improved detection sensitivity; (3) the lines are independent of each other to reduce cross interference; and (4) the line is only minimally influenced by external factors [22]. According to the above rules, the IR characteristic spectroscopy of the three target gases (CO, SO_2_ and H_2_S) is obtained by comparing the calculated and measured IR spectroscopic results, as shown in Table 4.

Comparing the calculated and measured IR spectroscopy of these three target gases, the calculated spectral intensity of CO and SO_2_ at their respective wavenumbers is 64.537 kJ/mol^−1^ and 188.75 kJ/mol^−1^, respectively; the calculated spectrum intensity of H_2_S is only 0.3246 kJ/mol^−1^ because the IR absorption capacity of CO and SO_2_ is much stronger than that of H_2_S gas. Due to these three target gases differing substantially in terms of IR absorption capacity, the experimental results show that CO and SO_2_ have distinct IR characteristic peaks with higher relative intensity but that the IR spectrum of H_2_S gas is noisier, with multiple IR peaks with lower relative intensity. It is important to ensure that the target detection gases exhibit strong and unambiguous IR characteristic peaks to enable reliable qualitative and quantitative detection of these target detection gases. Simulations based on the molecular dynamics calculated method are effective and help ensure the accuracy of the IR characteristic spectra of the target detection gases.

### 5.3. Pretreatment of IR Spectroscopy

IR absorption spectroscopy for quantitative detection is based on the Lambert-Beer Law. The quantitative analysis using this law is applied only in the absorbance spectrum. Therefore, the absorbance spectra of these target detection gases must be pretreated. The collected IR spectrum data may contain irrelevant information, such as noise, instrument interference, and environmental factors. The absorbance spectrum pretreatment can obtain a high-quality IR spectrum by eliminating this irrelevant information, thus improving the qualitative and quantitative accuracy.

**Figure 12:**
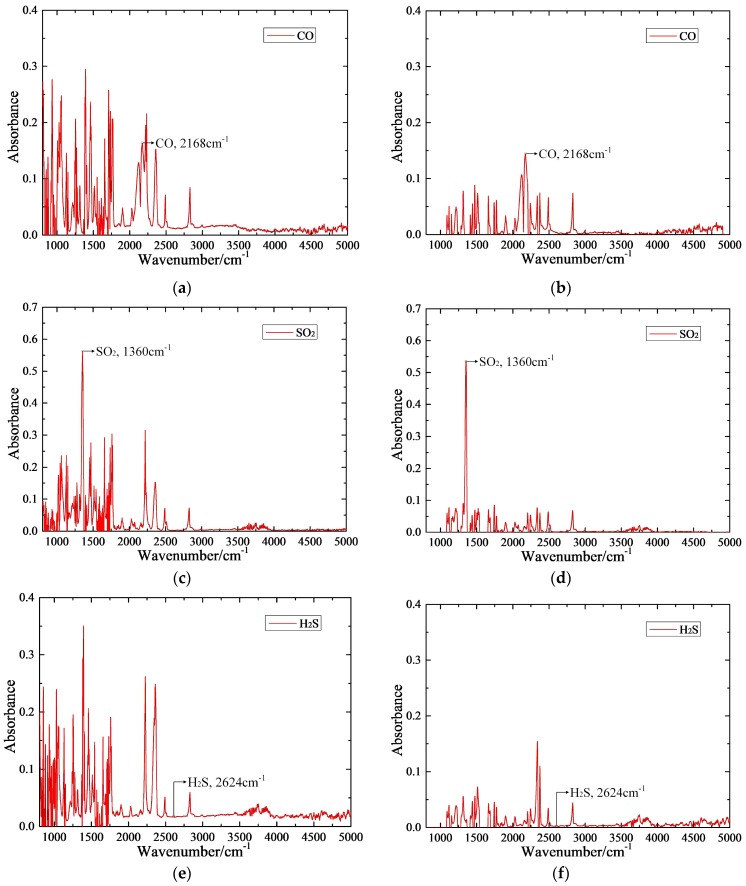
Pretreatment of the target gas IR spectrum. (**a**) CO before pretreating, (**b**) CO after pretreating, (**c**) SO_2_ before pretreating, (**d**) SO_2_ after pretreating, (**e**) H_2_S before pretreating, (**f**) H_2_S after pretreating.

Figure 12 shows that pretreatment of the target gas IR spectrum can fully retain the effective information by elimination of the interference noise and can further improve the quality of the absorbance spectrum of these target detection gases. Furthermore, the absorbance spectra of CO and SO_2_ gases feature prominent IR peaks at their characteristic wavenumber; thus, these two gases can be quantitatively detected based on their absorbance spectrum. Relative to that of CO and SO_2_, the IR absorption capacity of the H_2_S is far lower; H_2_S gas does not exhibit any prominent IR peak at its characteristic wavenumber and thus cannot be quantitatively detected using this FTIR detection system.

### 5.4. Response Characteristics of IR Spectroscopy

**Figure 13:**
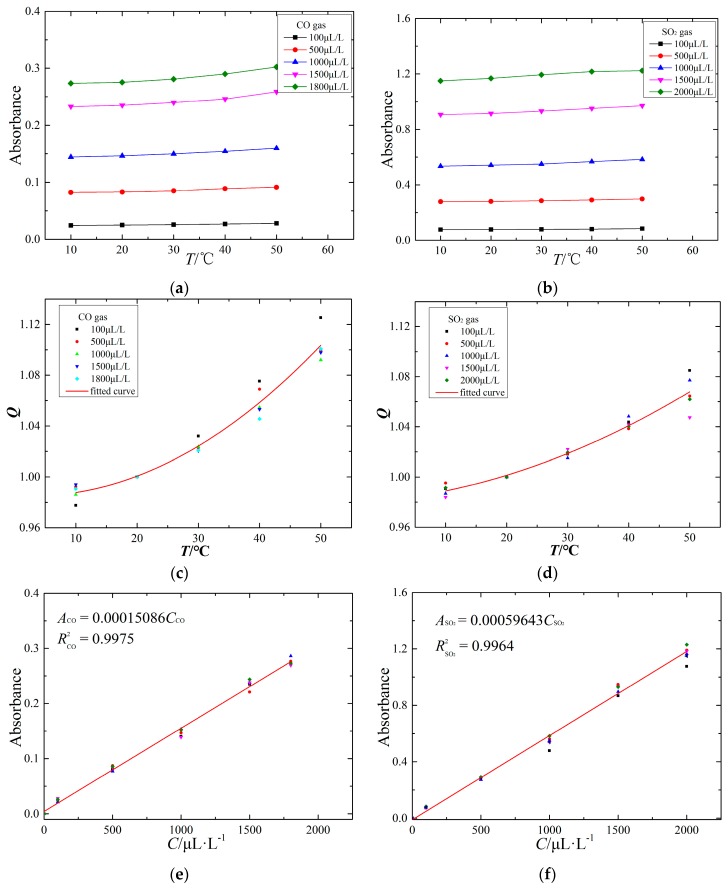
The response characteristics of the CO and SO_2_ IR spectrum: (**a**) temperature characteristic of CO IR spectrum; (**b**) temperature characteristic of SO_2_ IR spectrum; (**c**) temperature compensation of CO IR spectrum; (**d**) temperature compensation of SO_2_ IR spectrum; (**e**) linearity characteristic of CO IR spectrum; (**f**) linearity characteristic of SO_2_ IR spectrum.

Through analyzing the pretreatment absorbance spectrum of these three target detection gases, a prominent difference emerges between qualitative and quantitative detection. In the qualitative analysis, these three target gases (CO, SO_2_ and H_2_S) can all be identified using IR spectroscopy. In the quantitative analysis, CO and SO_2_ can be accurately calculated using the absorbance spectrum, but H_2_S gas is challenging to quantitatively analyze with IR spectroscopy, and a supplemental electrochemical sensor is required to realize quantitative analysis. However, CO and SO_2_ gas can be studied primarily in terms of the response characteristics IR absorption spectroscopy, as shown in Figure 13.

#### 5.4.1. Temperature Characteristics

The ambient temperature is an important factor influencing the measurement accuracy. To study the IR spectral characteristics of the target gas at different temperatures, the ambient temperature of the FTIR system is controlled using a vacuum oven. The temperature characteristics of the IR spectrum are obtained at 10 °C, 20 °C, 30 °C, 40 °C and 50 °C using the FTIR detection system to detect the experimental gas. The temperature characteristics are shown in Figure 13a,b.

Figure 13a,b shows that the absorbance value of CO and SO_2_ increases with increasing ambient temperature. Due to the IR absorption capacity of SO_2_ being stronger than that of CO, the SO_2_ absorbance value is larger than that of CO at the same concentration and ambient temperature. In general, increasing the ambient temperature may strengthen absorption of IR light by gas molecules; for example, the absorbance values of CO and SO_2_ increase with increasing temperature.

#### 5.4.2. Temperature Compensation

To further study the relationship between the temperature and IR absorbance of CO and SO_2_, the temperature compensation curves are fit by a quadratic fitting method, as shown in Figure 13c,d. The x-axis represents the ambient temperature (°C), and the *Q* of the y-axis represents the ratio of absorbance values at the measurement temperature and at 20 °C.

The absorbance spectra of CO and SO_2_ have a positive temperature relationship, and the change of ambient temperature influencing the CO absorbance value is much higher than that for SO_2_. The temperature compensation curves of CO and SO_2_ absorbance are analyzed as follows:

(14) $$\begin{array}{l} Q_{\mathrm{CO}}=0.98554-0.0003167T+0.00005351T^{2}\\ Q_{{\mathrm{SO}}_{2}}=0.98119+0.0005282T+0.00002412T^{2} \end{array}$$

where *Q* is the ratio of absorbance values at the measurement temperature and at 20 °C and *T* is the ambient temperature during a measurement.

#### 5.4.3. Linearity Characteristics

The quantitative analysis of IR spectroscopy follows the Lambert-Beer Law, and the absorbance value of the target gas is proportional to the target gas concentration. To study the linearity characteristics of the CO and SO_2_ IR spectra, through configuring various components and concentrations of experimental gas in the detection tank at one atmosphere and 20 °C ambient temperature, the linearity characteristics of CO and SO_2_ IR spectra are obtained, as shown in Figure 13e,f.

The experimental results show that the absorbance values of CO and SO_2_ follow a linear relationship with their gas concentration; the linearity characteristics of CO and SO_2_ IR spectra are represented by the following formulas:

(15) $$\begin{array}{l} A_{\mathrm{CO}}=0.00015086C_{\mathrm{CO}}\\ A_{{\mathrm{SO}}_{2}}=0.00059643C_{{\mathrm{SO}}_{2}} \end{array}$$

where *A* is the absorbance value of the target gas and *C* is the gas concentration.

Through analyzing the retreatment absorbance spectrum of CO and SO_2_, the signal-to-noise ratio (SNR) of their IR typical spectra can be obtained, and the theoretical detection precision of the FTIR detection system can be calculated. In addition, the upper envelope *A*_max_ = *k*_max_*C* and the lower envelope *A*_min_ = *k*_min_*C* are linearly fit using the least squares method. Comparing these two envelope lines with the linearity characteristics in Figure 13e,f, the measurement uncertainty can be calculated, as shown in Table 5.

### 5.5. Spectral Analysis Matrix

The IR absorption spectrum can directly and quickly reflect the gas components of the detected gas sample, and the gas concentration of each component can be quantitatively calculated based on the Lambert-Beer Law. To accurately obtain the target gas component and concentration, the spectral analysis matrix is assembled. The gas component can be quickly obtained by analyzing the IR spectrum. The absorbance value of the target gas is converted to the value at 20 °C, and the gas concentration is calculated based on the linearity characteristics of the CO and SO_2_ IR spectra:

(16) $$C=K^{-1}A=K^{-1}Q^{-1}A_{1}$$

where ***C*** = [*C*_CO_, *C*_SO_2__]*^T^* is the actual target gas concentration matrix, ***K*** = {*K_ij_*} (1 ≤ *i* ≤ 2, 1 ≤ *j* ≤ 2) represents the linearity characteristic matrices of the CO and SO_2_ IR spectra, ***Q*** = diag(*Q*_CO_, *Q*_SO_2__) represents the temperature compensation coefficient matrices of CO and SO_2_ IR spectra, ***A*** = [*A*_CO_, *A*_SO_2__,]*^T^* is the absorbance value matrix at 20 °C, and ***A*_1_** = [*A*_1CO_, *A*_1SO_2__]*^T^* is the absorbance value matrix for the actual measurement.

The multi-component gas mixture is used to verify the data matrix. Through configuring and detecting the multi-components with SF_6_ background gas with target gases of CO, SO_2_ and H_2_S, the experimental results of the FTIR detection system are shown in Table 6.

The experimental results show that the measurement uncertainty of the CO absorbance spectrum is less than 7% and that the measurement uncertainty of the absorbance spectrum is less than 5%.

## 6. ES-IR Comprehensive Detection

### 6.1. Analysis Model Based on ES-IR

ES and IR spectroscopy are important for SF_6_ decomposition analysis techniques for evaluating SF_6_ gas-insulated electrical equipment. These two detection methods are both suitable for online detection. In this section, ES and IR spectroscopy are integrated to detect SF_6_ gas decomposition through the response of electrochemical sensors and the spectral characteristics of target gases. A qualitative and quantitative analysis model for ES-IR has been established by adopting a two-step approach.

The first step is that the gas components of these three target gases (CO, SO_2_ and H_2_S) are quickly identified by analyzing the IR spectrum. Relative to the electrochemical sensor, IR spectroscopy is superior for qualitative analysis, offering a faster response speed, more potential gas detection components and less cross-interference, as shown in Table 7. Therefore, IR spectroscopy is used to rapidly identify the target gas components.

The second step is that the gas concentrations of these three target gases (CO, SO_2_ and H_2_S) are accurately calculated by analyzing the measurement results of these two methods. The joint detection algorithm is used to obtain the gas concentration. It is challenging to use a single measurement to accurately reflect the gas concentration; thus, this algorithm selects the average of five measurement results as the detection result (CES, CIR) of these two detection methods. In addition, the relative standard deviation of these five measurement results is chosen as the evaluation criteria, and the final result for the target gas can be calculated using the following equation:

(17) $$CV=\frac{\sqrt{\frac{\Sigma {\left(C_{i}-C_{o}\right)}^{2}}{n-1}}}{C_{o}}$$

where *CV* is the relative standard deviation of the five measurement results, *C*_o_ is the average of five measurement results, *C_i_* is a single measurement result, and *n* is the number of measurements. In addition:

(18) $$C=\frac{CV_{\mathrm{IR}}}{CV_{\mathrm{ES}}+CV_{\mathrm{IR}}}C_{\mathrm{ES}}+\frac{CV_{\mathrm{ES}}}{CV_{\mathrm{ES}}+CV_{\mathrm{IR}}}C_{\mathrm{IR}}$$

where *C* is the final result of the target gas, *CV*_ES_ is the relative standard deviation of the five measurement results using electrochemical sensors, *CV*_IR_ is the relative standard deviation of the five measurement results using IR spectroscopy, *C*_ES_ is the average of five measurement results using electrochemical sensors, and *C*_IR_ is the average of five measurement results using IR spectroscopy.

Thus, the qualitative and quantitative analysis model of ES-IR is established by adopting a two-step approach. The qualitative analysis uses IR spectroscopy to rapidly identify the gas components. The quantitative analysis uses a joint detection algorithm to calculate the measurement results of these two methods and then obtains the gas concentration of each identified component.

### 6.2. SF_6_ Decomposition Detector

**Figure 14:**
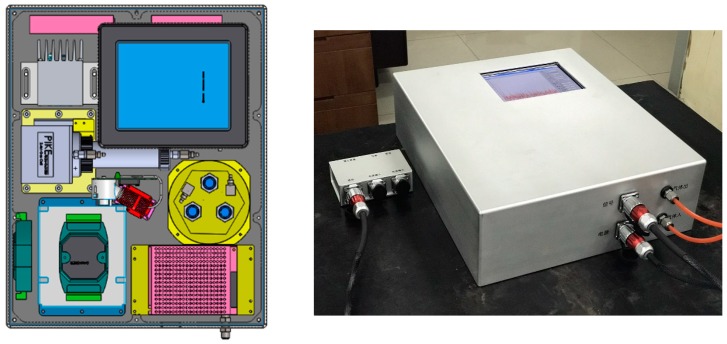
Structural design of the SF_6_ decomposition detector.

A SF_6_ decomposition detector was designed and manufactured by combining electrochemical sensors and IR spectroscopy, as shown in Figure 14. The volume of this detector is 460 mm × 380 mm × 132 mm, and the weight is 25 kg. Furthermore, there are several features for the developed SF_6_ decomposition detector in this paper. First of all, using ES-IR comprehensive detection method and cross interference technique, the gas composition and concentration of SF_6_ decomposition products could be analyzed accurately and fast, which meets the requirements of on-site monitoring. Secondly, temperature monitoring module is also installed inside the detector, then temperature compensation method is used to reduce the influence of ambient temperature on the measurement results and ensure the accuracy of the measurement results. Thirdly, pneumatic control module is applied to the detector to make the internal sensors work under the suitable condition of about 1 MPa gas pressure, which further ensures the veracity and reliability of test results.

The SF_6_ decomposition detector was tested using a multi-component gas mixture to verify the data matrix. Through configuring and detecting the multi-components with a background gas of SF_6_ and target gases of CO, SO_2_ and H_2_S, the results of the SF_6_ decomposition detector are shown in Table 8.

The results show that the measurement uncertainty for CO, SO_2_ and H_2_S is less than 5%. The detector is used to detect SF_6_ gas decomposition in the experiment, and the results show that the detector can reliably and accurately detect the gas components and concentrations.

## 7. Conclusions

ES and IR spectroscopy offer favorable application prospects for online detection. Furthermore, electrochemical sensors can be combined with IR spectroscopy to detect SF_6_ decomposition products; following this concept, a SF_6_ decomposition detector was also designed and manufactured. After reviewing the complete research results, the main conclusions are as follows:

(1) The SF_6_ decomposition detection method based on electrochemical sensors was studied. The results show that the sensors can accurately detect the three target gases (CO, SO_2_ and H_2_S). Through adopting temperature compensation and a crossing calculation, the sensor data matrix is established to guarantee measurement accuracy. A multi-component mixed gas is configured and used to verify the sensor data matrix, and the results show that the method offers satisfactory reliability and accuracy.
(2) Infrared spectroscopy is also an important detection method for analyzing SF_6_ decomposition. The IR absorption spectrum of SF_6_ and its decomposition products are simulated, and the typical IR spectra of the three target gases (CO, SO_2_ and H_2_S) were obtained. By assembling the spectral analysis matrix, the target gas can be rapidly identified, and CO and SO_2_ can also be quantitatively detected.
(3) The combination of electrochemical sensors with IR spectroscopy was used to detect SF_6_ gas decomposition. The qualitative and quantitative analysis model of ES-IR was established by adopting a two-step approach. A SF_6_ decomposition detector was designed and manufactured by combining electrochemical sensors and IR spectroscopy. The detector was used to detect SF_6_ gas decomposition in the experiments, and the results show that the detector can reliably and accurately detect the gas components and concentrations.

## Figures and Tables

**Table 1:** Linearity characteristics of the electrochemical sensors (at 20 °C).

Sensor	Target Gas	Slope (*m*)	Vertical Intercept (*n*)	Linearly Dependent Coefficient (*R*^2^)	Standard Error
CO	CO	8.1310	4	0.9934	0.0834
SO_2_	SO_2_	3.5571	4	0.9879	0.0541
	H_2_S	3.3049	4	0.9925	0.0875
H_2_S	H_2_S	12.735	4	0.9949	0.0690

**Table 2:** Theoretical detection precision and measurement uncertainty of the electrochemical sensors.

Sensors	Theoretical Precision (μL·L^−1^)	Measurement Uncertainty (%)	
		Upper Envelope	Lower Envelope
CO	1.2	2.94	−3.21
SO_2_	2.8	5.14	−4.16
H_2_S	0.8	3.24	−3.85

**Table 3:** The measurement results of SF_6_ decomposition products using a sensor data analysis matrix.

T (°C)	Sample (μL·L^−1^)			Measurement (μL·L^−1^)			Uncertainty (%)		
	CO	SO_2_	H_2_S	CO	SO_2_	H_2_S	CO	SO_2_	H_2_S
11.2	500	500	0	494	492	0	−1.20	−1.60	0
11.5	500	500	1000	500	514	1018	0.00	2.80	1.80
24.8	1000	500	0	985	495	0	−1.50	−1.00	0
25.1	1000	500	500	997	487	505	−0.30	−2.60	1.00
34.9	1000	1000	0	994	1027	0	−0.60	2.70	0
35.0	1000	1000	1000	983	1009	1030	−1.70	0.90	3.00
45.2	1500	1000	0	1480	983	0	−1.33	−1.70	0
45.4	1500	1000	500	1524	1016	503	1.60	1.60	0.60

**Table 4:** Typical IR spectra of the three target detection gases.

Detected Component	Characteristic Wavenumber (cm^−1^)	Spectroscopic Attribution	Calculated Intensity (kJ·mol^−1^)
CO	2168	C–O stretching vibration	64.537
SO_2_	1360	S=O symmetrical stretching vibration	188.75
H_2_S	2624	S–H stretching vibration	0.3246

**Table 5:** Theoretical detection precision and measurement uncertainty of IR absorption spectroscopy.

Electrochemical Sensor	Theoretical Precision (μL·L^−1^)	Measurement Uncertainty (%)	
		Upper Envelope	Lower Envelope
CO	4.8	7.87	−8.10
SO_2_	1.3	6.43	−7.24

**Table 6:** The measurement results for SF_6_ decomposition products using the spectral analysis matrix.

T (°C)	Sample (μL·L^−1^)			Measurement (μL·L^−1^)			Uncertainty (%)		
	CO	SO_2_	H_2_S	CO	SO_2_	H_2_S	CO	SO_2_	H_2_S
11.2	500	500	0	479	482	--	−4.20	−3.60	--
11.5	500	500	1000	526	514	--	5.20	2.80	--
25.0	1000	500	0	1036	490	--	3.60	−2.00	--
25.0	1000	500	500	1011	487	--	1.10	−2.60	--
35.0	1000	1000	0	930	952	--	7.00	−4.80	--
35.0	1000	1000	1000	972	1051	--	2.80	5.10	--
45.0	1500	1000	0	1450	979	--	−3.33	−2.10	--
45.4	1500	1000	500	1422	1016	--	−5.20	1.60	--

**Table 7:** Comparison of electrochemical sensors and IR spectroscopy in qualitative analysis.

Detection Method	Response	Gas Detection Components	Cross Interference
IR spectroscopy	1 spectrum/s	Most of the SF_6_ decomposition products (e.g., SO_2_F_2_, SOF_2_, CO, SO_2_, H_2_S, HF, CF_4_, and SiF_4_)	The typical spectrum of every component is independent
Electrochemical sensor	25–60 s	CO, SO_2_, H_2_S, and HF	Some components exhibit cross-interference

**Table 8:** The results of the SF_6_ decomposition detector.

No.	Sample (μL·L^−1^)			Measurement (μL·L^−1^)			Uncertainty (%)		
	CO	SO_2_	H_2_S	CO	SO_2_	H_2_S	CO	SO_2_	H_2_S
1	6.20	6.72	4.84	6	7	5	−3.23	4.17	3.31
2	11.99	12.41	9.55	12	13	10	0.08	4.75	4.71
3	24.34	19.03	20.21	24	19	20	−1.40	−0.16	−1.04
4	48.81	50.51	39.52	48	51	39	−1.66	0.97	−1.32
5	85.20	82.36	59.64	84	84	59	−1.41	2.00	−1.07
6	100	100	100	098	104	101	−2.00	4.00	1.00
7	500	500	100	500	488	103	0.00	−2.40	3.00
8	500	1000	500	514	1028	488	2.80	2.80	−2.40
9	1000	500	500	1034	484	504	3.40	−3.20	0.80
10	1000	1000	1000	1016	1047	1007	1.60	4.70	0.70

## Appendix A

**Table A1:** Main technical parameters of electrochemical sensors.

Sensor	Measuring Range (ppm)	Resolution Ratio (ppm)	Response Time (s)	Work Pressure (kPa)
CO	0–2000	0.5	<25	80–120
SO_2_	0–2000	1	<20	90–110
H_2_S	0–2000	−1	<60	90–110

**Table A2:** Main technical parameters of infrared spectrum detection system.

Module Name	Interferometer	Detector	IR Source	Gas Pool
Main technical parameters	Type: Dual reflector design	Type: Photoelectric MCT	Type: ceramic lamp filament	Type: Gas Cell-2.4 m Optical path length: 2.4 m
	Lens diameter: 12.7 mm	Aperture diameter: 12.7 mm	Beam diameter: 12.7 mm	
	Resolution ratio: 4 cm^−1^	Resolution ratio: 1 mm	Spectrum range: 1–25 μm	
	Repetition rate of wave number: <10 ppm	Amplifier bandwidth: 0–20 kHz	Power source: 12/5 A	
	Scanning frequency: 1 spectrum per second	Half-field angle: 28 mrad	Scanning frequency: 1 spectrum per second	

**Table A3:** Comparison between calculated and measured IR spectroscopy.

Decomposition Products	Simulation (cm^−1^)	Measurement (cm^−1^)	Relative Error (%)
SO_2_F_2_	525.15; 532.25; 540.90; 821.17; 1278.56; 1526.79	539; 544; 552; 848; 1269; 1502	−2.57; −2.16; −2.01; −3.16; 0.75; 1.65
SOF_2_	373.25; 511.97; 780.89; 1355.55	390; 530; 806; 1333	−4.29; −3.40; −3.12; 1.69
CO	2086.39	2169	−3.81
SO_2_	1163.69; 1368.85	1167; 1360	−0.28; 0.65
H_2_S	1380.97; 2657.83	1335; 2625	3.44; 1.25
HF	3621.93	3644	−0.61
CF_4_	1231.598; 1232.343	1280; 1283	−3.78; −3.95
SiF_4_	1044.98; 1045.93; 1047.79	1020; 1028; 1032	2.45; 1.74; 1.53
